# Supplementary material for: Interplay between midbrain and dorsal anterior cingulate regions arbitrates lingering reward effects on memory encoding
Source: Nat Commun. 2020 Apr 14;11:1829. doi: 10.1038/s41467-020-15542-z (PMC7156375; doi:10.1038/s41467-020-15542-z)
Supplement: Supplementary file 1 — Supplementary Information [file 41467_2020_15542_MOESM1_ESM.pdf]

Supplementary information

Interplay between midbrain and dorsal anterior cingulate regions arbitrates lingering reward effects on memory encoding

KC Aberg, EE Kramer, S Schwartz

Supplementary analyses

Behavior

Supplementary Note 1: Controlling for encoding and testing positions and character identity on memory performance

To ensure that the observed behavioral results, i.e. the main effects of Feedback value and Average reward, are not confounded by the identity of a particular cartoon character, or the position in which a particular association was encoded or tested, we performed an additional analysis controlling for these potentially confounding factors. Specifically, in a linear mixed-effects model we included the trial number in which an association was encoded, the identity of the cartoon character, and the trial in which the association was tested. The rationale for including these factors-of-no-interest in the model is that any shared variance with the factors-of-interest (i.e. Feedback value and Average reward category) is removed from the analysis. Accordingly, if the variance assigned to the factors-of-interest can be (partially) explained by the factors-of-no-interest, the factors-of-interest should no longer be significant. However, the main effects of feedback value and average reward were remained significant also in this new model [Feedback value: $F(2, 1945) = 22.28$, $p<0.001$; Average reward: $F(2, 1945)=3.55$, $p=0.029$, ANOVA], while their interaction was not significant [$F(4, 1945)=1.53$, $p=0.191$, ANOVA]. In other words, the impact of feedback value and average reward on memory encoding did not depend on where in a sequence of trials an association was encoded, nor did it depend on the identity of the cartoon character.

Computational modeling

Supplementary Note 2: Fitting different parameters for different cartoon characters

The extended analysis of behavioral data (see previous paragraph) suggested that the character identity did not modulate the main effects of Feedback value and Average reward. To further validate this null-result, we also used a computational approach to test whether fitting character-

specific parameters improved the model-fit to behavior. Two models, based on the most parsimonious model was thus created. As a reminder, for the most parsimonious model the current reward was defined as:

$$R_{fb+\bar{r}^\wedge 2}(t) = C_0 + C_{fb} * r(t) + C_{\bar{r}^\wedge 2} * (\bar{r}(t) - w))^2$$

Average reward is calculated as an exponential running average with learning rate v:

$$\bar{r}(t) = v * fb(t) + (1 - v) * \bar{r}(t - 1)$$

The probability of encoding an association was defined as:

$$p_E(t) = \frac{1}{1 + e^{-R(t)}}$$

Now, to test whether characters may be associated with different initial levels of average reward (i.e. $\boldsymbol{C_{\bar{r}0}}$), a new model was designed in which six additional parameters (i.e. one $\boldsymbol{C_{\bar{r}0}}$ for each character) were fitted to behavior, in addition to the parameters of the most parsimonious model. Another new model tested whether characters may be associated with different rates of accumulating average reward (i.e. different learning rates v). Thus, six different v (i.e. one for each character) replaced the single v of the most parsimonious model and were fitted to behavior.

The results of these fits are shown in Supplementary Table 1. None of these two new models provided a better fit to data, as compared to the most parsimonious model [mean AIC parsimonious model=107.67±2.93 versus i) mean AIC inter-character initial average reward=115.07±2.65, *t*(32)=4.439, p<0.001, paired *t*-test; ii) mean AIC inter-character learning rates=113.85±2.54, *t*(32)=4.575, p<0.001, paired *t*-test].

Supplementary table 1. Model fits.

Model	$R_{fb+\bar{r}^2}$*	$R_{fb+\bar{r}^2}$ with inter-character initial average reward	$R_{fb+\bar{r}^2}$ with inter-character learning rates
**LLE**	48.84±1.46	46.53±1.33	46.93±1.27
**AIC**	107.67±2.93	115.07±2.65	113.85±2.54
C_0	0.44±0.09	0.72±0.07	0.53±0.08
C_{fb}	0.61±0.10	0.61±0.10	0.62±0.10
$C_{\bar{r}^2}$	-42.73±12.47	-3.14±0.57	-4.39±0.77
w	0.43±0.09	0.18±0.13	0.34±0.11
v	0.44±0.07	0.27±0.06	-
$C_{\bar{r}01}$	-	0.50±0.07	-
$C_{\bar{r}02}$	-	0.38±0.06	-
$C_{\bar{r}03}$	-	0.46±0.07	-
$C_{\bar{r}04}$	-	0.48±0.07	-
$C_{\bar{r}05}$	-	0.47±0.07	-
$C_{\bar{r}06}$	-	0.58±0.07	-
v_1	-	-	0.48±0.07
v_2	-	-	0.49±0.07
v_3	-	-	0.48±0.07
v_4	-	-	0.40±0.07
v_5	-	-	0.50±0.07
v_6	-	-	0.47±0.07

LLE is the negative log-likelihood estimate. AIC is Akaike's Information Criterion. C_0, C_{fb}, $C_{\bar{r}0}$, $C_{\bar{r}^2}$, v, and w are parameters fitted to each individual's behavior. $C_{\bar{r}0i}$denotes the initial average reward level for character *i*. v_i denotes the learning rate for character *i*. * Denotes the model providing the most parsimonious fit to behavior, as indicated by significantly lower AIC values. Mean ± SEM.

Supplementary Note 3: Model-predicted memory performance

To test whether model-predicted behavior matched actual behavior, the individually fitted parameters of the most parsimonious model was used to calculate an encoding probability in each trial for each participant. As with actual behavior, a linear mixed effects model was fitted to behavior with fixed effects Feedback value (-1, +1, +5) and Average reward (Lo, Me, Hi) and participant as random effect. This analysis revealed a significant main effect of Feedback value [Figure 2E main text; F(2, 288)=61.640, p<0.001, ANOVA] because better memory was predicted for character-object associations encoded during +5 feedback [mean±SEM: 0.724±0.023] as compared to +1 feedback [mean±SEM: 0.647±0.019, *t*(32)=5.205, p<0.001, d=0.639, 95% CI=0.047-0.107, paired *t*-test] and -1 feedback [mean±SEM: 0.587±0.018, *t*(32)=6.616, p<0.001, d=1.179, 95% CI=0.095-0.180, paired *t*-

test]. Associations encoded during +1 feedback were also better remembered as compared to -1 feedback [*t*(32)=5.046, p<0.001, d=0.578, 95% CI=0.036-0.085, paired *t*-test].

The main effect of Average reward was also significant [Figure 2F main text; F(2,288)=11.636, p<0.001 ANOVA], with highest memory performance predicted for associations encoded during Me average reward [mean±SEM: 0.686±0.019], both as compared to Lo average reward [mean±SEM: 0.644±0.021, *t*(32)=3.644, p<0.001, d=0.357, 95% CI=0.018-0.065, paired *t*-test], and Hi average reward [mean±SEM: 0.628±0.019, *t*(32)=3.915, p<0.001, d=0.357, 95% CI=0.028-0.089, paired *t*-test]. There was no difference in predicted memory performance between Lo and Hi average reward [*t*(32)=871, p=0.390, d=0.146, 95% CI=-0.022-0.056, paired *t*-test]. The interaction between Feedback value and Average reward was not significant [F(4,288)=0.850, p=0.495, ANOVA]. In summary, these results suggest that the most parsimonious model captures relevant aspects of memory performance as a function of feedback value and average reward.

fMRI

Supplementary Note 4: A ROI based analysis of VTA activation by feedback value

Initially, we tested VTA activation using an anatomically defined VTA ROI[1] using a strict voxel selection criteria (only voxels shared by at least 50% of participants were included), and found no significantly activated voxels in the VTA ROI by the parametric modulator feedback value. To test whether this null-result might be related to the rather strictly and anatomically defined ROI, we performed a conservative ROI analysis, by calculating the average beta parameter estimate across all voxels, within another functionally defined VTA ROI which we previously used to test midbrain encoding of prediction errors [2]. This ROI was created by centering two 4mm radius spheres on the Tailarach coordinates [-4 -15 -9] and [5 -14 -8], obtained from a previous study testing reward-related memory enhancements[3], transformed to MNI space (Supplementary Figure 1A). Indeed, on average, the BOLD signal of voxels within this VTA ROI correlated significantly with feedback value

(Supplementary Figure 1B; mean beta parameter estimate±SEM=0.171±0.083, $t(32)=2.066$, p=0.024, d=0.470, 95% CI=0.031-Infinity, one-tailed *t*-test).

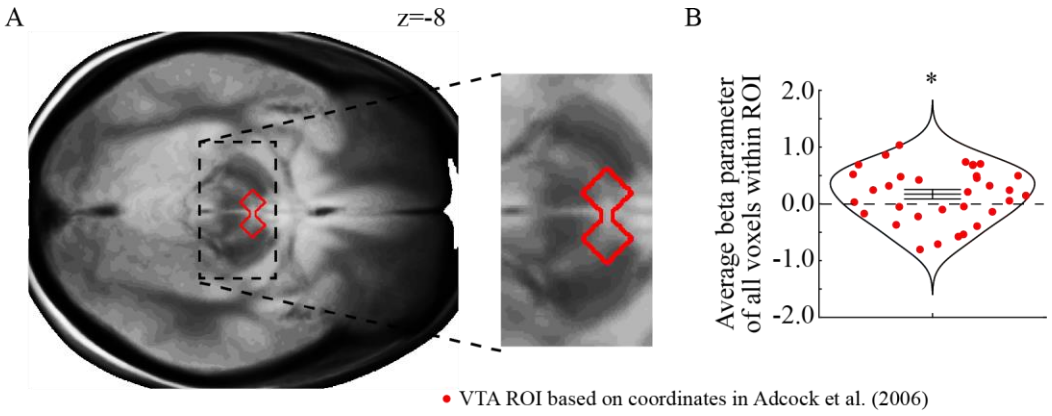

Supplementary Figure 1. A. Visual representation of the tested VTA ROI. B. Violin plot showing the average beta parameter estimate for the modulation by Feedback value extracted from the ROI. The average beta parameter estimate was significantly above 0.0. The horizontal lines indicate mean±SEM. * $p < 0.05$, indicates the p-value for paired *t*-tests. Source data are provided as a Source Data file.

Supplementary Note 5: Robustness of the fMRI correlates of average reward in the midbrain

Pivotal to the present study is that average reward was represented in an inverted U-shape fashion in the VTA. To confirm the robustness of this result, we performed additional analyses in which we tested the VTA activation using a more conservative ROI approach in which the activity of all voxels within an a priori ROI is averaged and compared to the null-hypothesis of no significant activation (i.e. a beta parameter estimate of 0.0). This analysis was performed for two different ROIs and for two different smoothing kernels. The first ROI (i.e. identical to the VTA ROI used in the main text) was obtained from a probabilistic atlas of the VTA [1], but restricted to voxels shared by 50% of the participants. The second ROI was based on coordinates obtained from a previous study investigating reward-related memory enhancements [3]. This ROI was created by centering two 4mm radius spheres on the Tailarach coordinates [-4 -15 -9] and [5 -14 -8] transformed to MNI space. We previously used this approach to analyze the neural representation of prediction errors pertaining to the dopaminergic midbrain [2]. The first smoothing kernel was identical to the one used in the main text (i.e. 8mm), but we also tested the results when using a 4mm smoothing kernel which may be more appropriate when assessing smaller structures, such as the VTA. The different ROIs are shown in Supplementary Figure 2A, and the average extracted beta parameters are shown in Supplementary Figure 2B. For all combinations of ROIs and smoothing kernels, the BOLD signal was significantly activated by average reward in an inverted U-shape fashion [Murty ROI + 8mm smoothing: mean beta parameter estimate±SEM=-0.283±0.118, $t(32)=2.392$, p=0.023, d=0.589, 95% CI=-0.523--0.042; Adcock ROI + 8mm smoothing: mean beta parameter estimate±SEM=-0.446±0.124, $t(32)=3.593$, p=0.001, d=0.885, 95% CI=-0.699--0.193, Murty ROI + 4mm smoothing: mean beta parameter estimate±SEM=-0.315±0.130, $t(32)=2.428$, p=0.021, d=0.598, 95% CI=-0.579--0.051; Adcock ROI + 4mm smoothing: mean beta parameter estimate±SEM=-0.497±0.142, $t(32)=3.502$, p=0.001, d=0.862, 95% CI=-0.786--0.208, all p-values obtained via two-tailed *t*-tests].

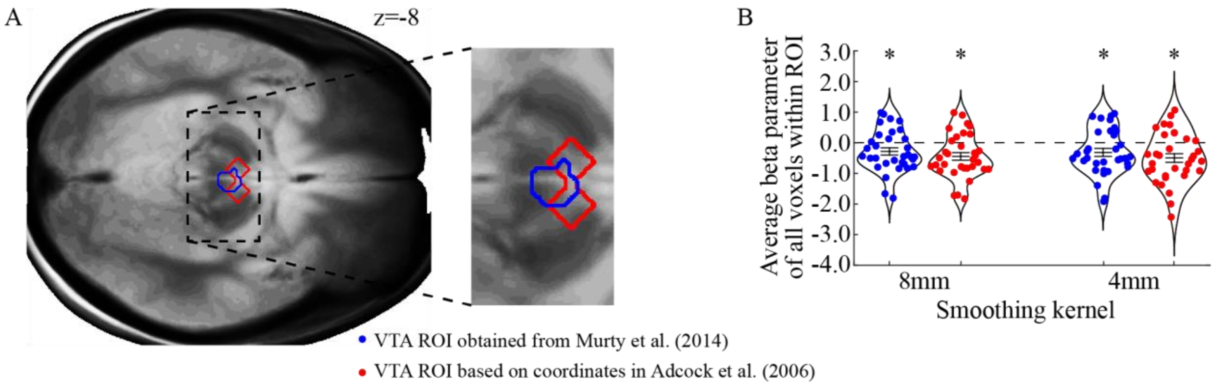

Supplementary Figure 2. A. Visual representation of the two tested VTA ROIs. B. Violin plots showing the average beta parameter estimate for the modulation by average reward (inverted U-shape) extracted from the two different ROIs and the two different smoothing kernels. In all four cases, the average beta parameter was significantly below 0.0. The horizontal lines indicate mean±SEM. * $p < 0.05$, indicates the p-value for paired *t*-tests. Source data are provided as a Source Data file.

Supplementary Note 6: Sagittal views of BOLD signal correlates of feedback value and average reward (inverted U-shape)

To provide a more detailed illustration of reward-related neuronal activation in the temporal lobe, Supplementary Figure 3 contains sagittal views of BOLD signal in the temporal lobe that correlates with Feedback value and Average reward (inverted U-shape).

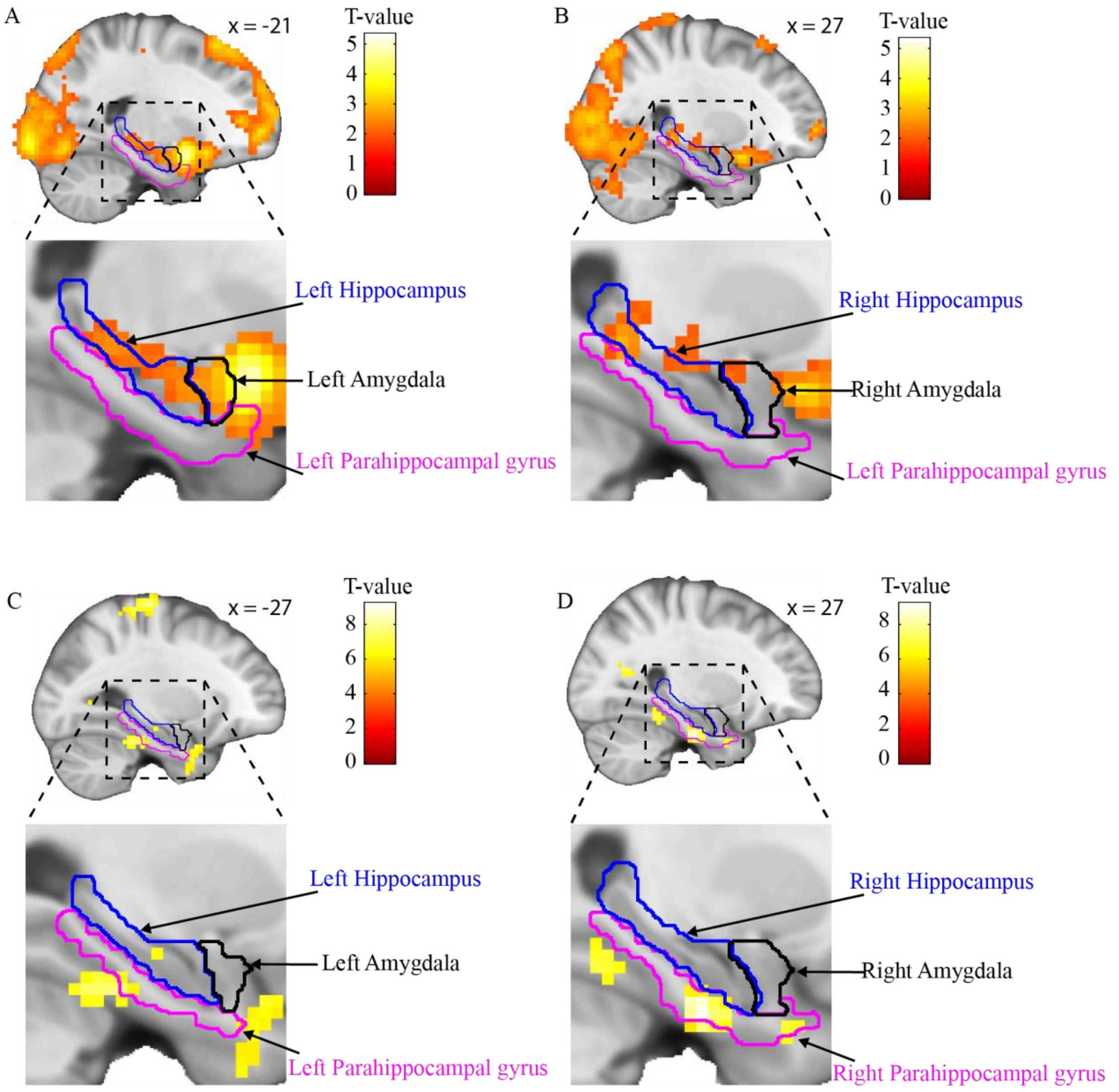

Supplementary Figure 3. Sagittal views of BOLD signal correlations with Feedback value and Average reward (inverted U-shape). BOLD signal correlating with Feedback value in the left (A) and the right (B) hemisphere. BOLD signal correlating with Average reward (inverted U-shape) in the left (C) and the right (D) hemisphere. Activations are displayed using an uncorrected threshold of p=0.001 (obtained from *t*-test).

Supplementary Note 7: fMRI correlates of feedback value and average reward during stimulus onset

Reward-anticipation is evoked by the presentation of stimuli previously associated with rewards [4], and enhances memory encoding via reward circuitry [3, 5]. Thus, the impact of average reward on memory formation could (at least in part) be related to neuronal activity evoked at the presentation

of character-object pairs. For this reason, we tested whether BOLD signal at stimulus onset correlated with feedback value and the inverted U-shape function of average reward. In brief, this event-related design included three events (stimulus onset, response, and feedback onset) modeled as stick functions (i.e. with zero duration). Model-derived feedback-values and average reward were then added as parametric regressors to the stimulus onset, rather than the feedback onset as in the original design. In brief, no voxels within our pre-defined ROIs correlated with these parametric modulators. To illustrate this null-result, all significantly activated voxels are shown in a glass-brain using an uncorrected threshold of p=0.001, and then all significant voxels within our a priori masks (including the bilateral amygdala, nucleus accumbens, hippocampus, parahippocampal gyrus, dorsal anterior cingulate cortex, and the ventral tegmental area) are shown in a glass-brain using an uncorrected threshold of p=0.05. The results for the feedback value is shown in Supplementary Figure 4A,B while the results for average reward is shown in Supplementary Figure 4C,D. Clearly, no voxels show significant modulation by feedback value and average reward within a priori ROIs, therefore suggesting that neuronal activation at stimulus onset does not contribute to the observed effects of average reward on memory formation.

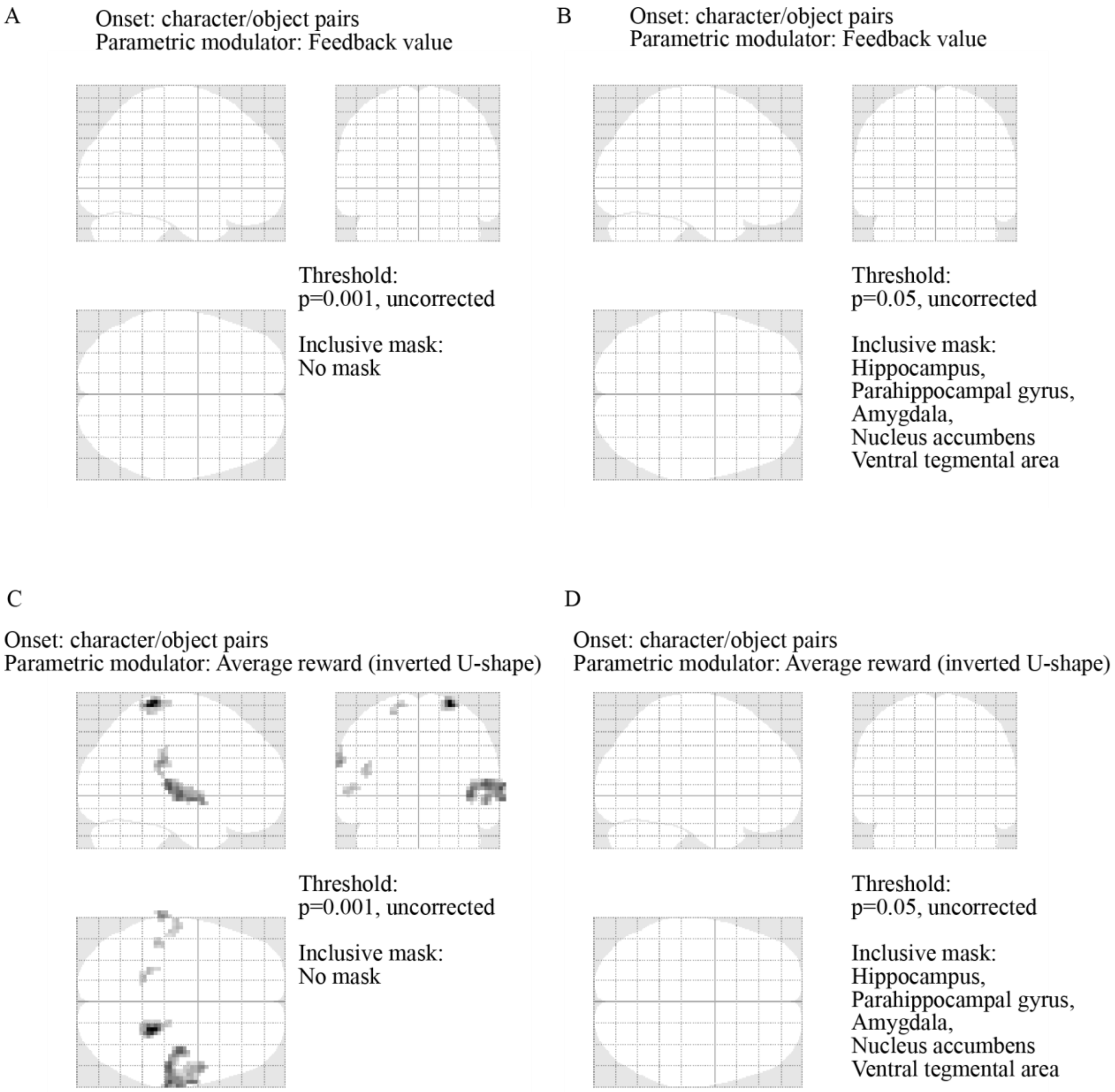

Supplementary Figure 4. Glass brains showing BOLD signal at the onset of character-object pairs. A. Parametric modulation by Feedback value at uncorrected threshold p=0.001, no inclusive mask. B. Parametric modulation by Feedback value at uncorrected threshold p=0.05, inclusively masked by the hippocampus, parahippocampal gyrus, amygdala, ventral tegmental area, and the nucleus accumbens. C. Parametric modulation by Average reward (inverted U-shape) at uncorrected threshold p=0.001, no inclusive mask. D. Parametric modulation by Average reward (inverted U-shape) at uncorrected threshold p=0.05, inclusively masked by the hippocampus, parahippocampal gyrus, amygdala, ventral tegmental area, and the nucleus accumbens. All p-values were obtained from *t*-tests.

Supplementary references

1. Murty VP, Shermohammed M, Smith DV, Carter RM, Huettel SA, Adcock RA. Resting state networks distinguish human ventral tegmental area from substantia nigra. *Neuroimage* **100**, 580-589 (2014).

2. Aberg KC, Doell KC, Schwartz S. Hemispheric Asymmetries in Striatal Reward Responses Relate to Approach-Avoidance Learning and Encoding of Positive-Negative Prediction Errors in Dopaminergic Midbrain Regions. *The Journal of neuroscience : the official journal of the Society for Neuroscience* **35**, 14491-14500 (2015).

3. Adcock RA, Thangavel A, Whitfield-Gabrieli S, Knutson B, Gabrieli JD. Reward-motivated learning: mesolimbic activation precedes memory formation. *Neuron* **50**, 507-517 (2006).

4. Schultz W, Carelli RM, Wightman RM. Phasic dopamine signals: from subjective reward value to formal economic utility. *Current opinion in behavioral sciences* **5**, 147-154 (2015).

5. Wittmann BC, Schott BH, Guderian S, Frey JU, Heinze HJ, Duzel E. Reward-related FMRI activation of dopaminergic midbrain is associated with enhanced hippocampus-dependent long-term memory formation. *Neuron* **45**, 459-467 (2005).
