# Supplementary material for: Interplay between midbrain and dorsal anterior cingulate regions arbitrates lingering reward effects on memory encoding
Source: Nat Commun. 2020 Apr 14;11:1829. doi: 10.1038/s41467-020-15542-z (PMC7156375; doi:10.1038/s41467-020-15542-z)
Supplement: Supplementary file 3 — Reporting Summary [file 41467_2020_15542_MOESM3_ESM.pdf]

nature research

Corresponding author(s): Kristoffer Aberg
kc.aberg@gmail.com

Last updated by author(s): Jan 29, 2020

Reporting Summary

Nature Research wishes to improve the reproducibility of the work that we publish. This form provides structure for consistency and transparency in reporting. For further information on Nature Research policies, see Authors & Referees and the Editorial Policy Checklist.

Statistics

For all statistical analyses, confirm that the following items are present in the figure legend, table legend, main text, or Methods section.

n/a	Confirmed	
☐	☒	The exact sample size (*n*) for each experimental group/condition, given as a discrete number and unit of measurement
☐	☒	A statement on whether measurements were taken from distinct samples or whether the same sample was measured repeatedly
☐	☒	The statistical test(s) used AND whether they are one- or two-sided<br>*Only common tests should be described solely by name; describe more complex techniques in the Methods section.*
☒	☐	A description of all covariates tested
☐	☒	A description of any assumptions or corrections, such as tests of normality and adjustment for multiple comparisons
☐	☒	A full description of the statistical parameters including central tendency (e.g. means) or other basic estimates (e.g. regression coefficient) AND variation (e.g. standard deviation) or associated estimates of uncertainty (e.g. confidence intervals)
☐	☒	For null hypothesis testing, the test statistic (e.g. *F*, *t*, *r*) with confidence intervals, effect sizes, degrees of freedom and *P* value noted<br>*Give P values as exact values whenever suitable.*
☒	☐	For Bayesian analysis, information on the choice of priors and Markov chain Monte Carlo settings
☒	☐	For hierarchical and complex designs, identification of the appropriate level for tests and full reporting of outcomes
☐	☒	Estimates of effect sizes (e.g. Cohen's *d*, Pearson's *r*), indicating how they were calculated

Our web collection on statistics for biologists contains articles on many of the points above.

Software and code

Policy information about availability of computer code

Data collection	Matlab 2016b + Psychtoolbox version 3.0 for Matlab.
Data analysis	Matlab 2016b + Matlab Statistical toolbox. + SPM version 8 with the gPPI, WFU, and Anatomy toolboxes for SPM.

For manuscripts utilizing custom algorithms or software that are central to the research but not yet described in published literature, software must be made available to editors/reviewers. We strongly encourage code deposition in a community repository (e.g. GitHub). See the Nature Research guidelines for submitting code & software for further information.

Data

Policy information about availability of data

All manuscripts must include a data availability statement. This statement should provide the following information, where applicable:

- Accession codes, unique identifiers, or web links for publicly available datasets
- A list of figures that have associated raw data
- A description of any restrictions on data availability

The data that support the findings of this study are available from the corresponding author upon reasonable request. Raw data are provided in a separate source-file for the following Figures:
1E,1F
2E, 2F, 2G
3B, 3C, 3E, 3F, 3H, 3I
4B, 4D, 4E, 4G, 4H
5C, 5E
Supplementary Figures 1B and 2B

Field-specific reporting

Please select the one below that is the best fit for your research. If you are not sure, read the appropriate sections before making your selection.

- [ ] Life sciences
- [x] Behavioural & social sciences
- [ ] Ecological, evolutionary & environmental sciences

For a reference copy of the document with all sections, see nature.com/documents/nr-reporting-summary-flat.pdf

Behavioural & social sciences study design

All studies must disclose on these points even when the disclosure is negative.

Study description	This is a quantitative fMRI study on human memory.
Research sample	We tested 34 students at Geneva University (mean age 25 years, 15 females). All participants were right-handed, native French speakers, and without any previous history of psychiatric or neurological disorders.
Sampling strategy	Participants were selected randomly. The number of participants was determined based on the results of a previous behavioral study and an appropriate sample size for fMRI.
Data collection	Data collection took place inside a Siemens 3T MRI scanner. The conditions were equal for all participants. MRI technicians and one researcher was always present inside the control room during data collection.
Timing	Data collection started 09.04.2014 and ended 24.10.2014.
Data exclusions	Data from one person was excluded from the data analysis because he/she feel asleep during the memory test phase.
Non-participation	All recruited participants performed the experiment.
Randomization	The was no randomization procedure needed.

Reporting for specific materials, systems and methods

We require information from authors about some types of materials, experimental systems and methods used in many studies. Here, indicate whether each material, system or method listed is relevant to your study. If you are not sure if a list item applies to your research, read the appropriate section before selecting a response.

Materials & experimental systems

n/a	Involved in the study	
☒	☐	Antibodies
☒	☐	Eukaryotic cell lines
☒	☐	Palaeontology
☒	☐	Animals and other organisms
☐	☒	Human research participants
☒	☐	Clinical data

Methods

n/a	Involved in the study	
☒	☐	ChIP-seq
☒	☐	Flow cytometry
☐	☒	MRI-based neuroimaging

Human research participants

Policy information about studies involving human research participants

Population characteristics	See above.
Recruitment	Participants were recruited via advertisements on Geneva University campuses.
Ethics oversight	Geneva University Hospital.

Note that full information on the approval of the study protocol must also be provided in the manuscript.

Magnetic resonance imaging

Experimental design

Design type	We used an event-related design.

Design specifications	All participants performed 120 trials. Each trial lasted on average 8 seconds and trials were separated by an average of 3 seconds.
Behavioral performance measures	We recorded button presses and reaction times. Behavioral performance was determined by calculating proportion correct, and reaction times were used to determine if a person had fallen asleep (if they repeatedly failed to elicit a response with 3.5 seconds after a stimulus had been presented). Data from one person was excluded for this reason.

Acquisition

Imaging type(s)	Functional and structural.
Field strength	3T
Sequence & imaging parameters	MRI images were acquired using a 3T whole body MRI scanner (Trio TIM, Siemens, Germany) with a 12-channel head coil. Standard structural images were acquired with a T1 weighted 3D sequence (MPRAGE, TR/TI/TE = 1900/900/2.27 ms, flip angle = 9 degrees, voxel dimensions = 1 mm isotropic, 256 x 256 x 192 voxels). Proton density (PD) structural images were acquired with a turbo spin echo sequence (TR/TE = 6000/8.4 ms, flip angle = 149 degrees, voxel dimensions = 0.8x0.8x3 mm, 205 x 205 x 60 voxels). The PD scan was used to confirm the location of VTA activation, as it allows the identification of the substantia nigra, a brain region located just laterally to the VTA 36. The acquisition volume was oriented in order to scan the brain from the lower part of the pons to the top of the thalamus. Functional images were acquired with a susceptibility weighted EPI sequence (TR/TE = 2100/30 ms, flip angle = 80 degrees, voxel dimensions = 3.2 mm isotropic, 64 x 64 x 36 voxels).
Area of acquisition	Whole brain scanning was used for fMRI and standard structural imaging, while the PD scan focused on the VTA (as outlined above).

Diffusion MRI ☐ Used ☒ Not used

Preprocessing

Preprocessing software	Functional MRI data were preprocessed and then analyzed using the general linear model (GLM) for event-related designs in SPM8 (Welcome Department of Imaging Neuroscience, London, UK; http://www.fil.ion.ucl.ac.uk/spm). During preprocessing, all functional volumes were realigned to the mean image, co-registered to the structural T1 image, corrected for slice timing, normalized to the MNI EPI-template, and smoothed using an 8 mm FWHM Gaussian kernel.
Normalization	Normalization was performed using standard procedures implemented in SPM8, and by using mainly default settings. Normalization included a 12 parameter affine transformation model and a non-linear transformation model using a set of cosine basis functions. The voxel size was changed from the default setting of [2 2 2] mm to the actual voxel size of [3 3 3.5] mm.
Normalization template	SPM8's EPI template [3 3 3] mm.
Noise and artifact removal	Six rigid-body realignment parameters were included as nuisance covariates when estimating statistical maps.
Volume censoring	No volume censoring was performed.

Statistical modeling & inference

Model type and settings	At the first level, we used an event-related design that included two event-types that were respectively time-locked to the onset of stimuli and feedback, respectively, in each trial. We added trial-by-trial estimates of model-derived parameters as parametric modulators to the feedback onset times. On the second level, we used paired t-tests implemented in SPM, and applied appropriate corrections for multiple comparisons.
Effect(s) tested	Using t-tests, we tested for brain regions showing a statistical relationship with model-derived parametric modulators, specifically trial-wise changes in reward feedback and average reward.

Specify type of analysis: ☐ Whole brain ☐ ROI-based ☒ Both

Anatomical location(s)	Anatomical brain regions were determined based on previous related research. Their locations were obtained from the WFU toolbox, the Anatomy toolbox, and a probabilistic atlas was used to define the VTA.
Statistic type for inference (See Eklund et al. 2016)	We used peak-voxel inference corrected for multiple comparisons within a prioi ROIs. The search threshold was set to p=0.001 and a minimal cluster size of ten contiguous voxels.
Correction	We used FWE correction for multiple comparisons.

Models & analysis

n/a	Involved in the study
☐	☒ Functional and/or effective connectivity
☒	☐ Graph analysis
☒	☐ Multivariate modeling or predictive analysis

Functional and/or effective connectivity

We report psychophysiological interactions (PPI) between the VTA (source region) and the dACC.
